# Multidisciplinary Management of an Internal Carotid Artery Aneurysm Near the Skull Base

**DOI:** 10.7759/cureus.62086

**Published:** 2024-06-10

**Authors:** Sameer Kejriwal, Hannah Bulosan, Nicolas A Nelken, Jae H Lim

**Affiliations:** 1 School of Medicine, University of Hawaii John A. Burns School of Medicine, Honolulu, USA; 2 Department of Vascular Surgery, Hawaii Permanente Medical Group, Kaiser Moanalua Medical Center, Honolulu, USA; 3 Department of Otolaryngology-Head and Neck Surgery, Hawaii Permanente Medical Group, Kaiser Moanalua Medical Center, Honolulu, USA

**Keywords:** multidisciplinary, extracranial, carotid bypass, skull base, internal carotid artery aneurysm

## Abstract

Extracranial carotid artery aneurysms (ECAAs) are rare in comparison to the total number of peripheral artery aneurysms. Although there are multiple treatment modalities, no clear guidelines exist for the optimal management of ECAA. We describe a case of a 59-year-old female with an incidental finding of a 2.6 cm right internal carotid artery (ICA) aneurysm on computed tomography (CT) that was eventually excised via transcervical approach followed by end-to-end anastomosis with great saphenous vein (GSV) graft. To our knowledge, this case demonstrates a novel multidisciplinary approach to an ECAA near the skull base involving head and neck surgery (HNS), vascular surgery (VS), and neuro-interventional radiology (NIR).

## Introduction

Extracranial carotid artery aneurysms (ECAAs) are rare, accounting for less than 1% of all arterial aneurysms [1,2]. Atherosclerosis has been described as a main etiological factor of ECAA [3,4]. Other causes include trauma, infections, prior irradiation, and connective tissue disorders [3,4]. Patients may be asymptomatic, with discovery as an incidental finding, or present with symptoms such as a pulsatile neck mass, cerebral ischemia, or cranial nerve deficits [3]. When left untreated, ECAAs have reported neurological events as high as 50% [3]. Treatment options include medical management, endovascular intervention, and open surgery [1,5]. Given the increased long-term risks with only medical treatment, management for symptomatic patients tends to be between endovascular intervention and open surgery [5]. However, no clear guideline exists regarding optimal management. Therefore, the management of ECAA depends on factors such as aneurysm size, location, etiology, and patient comorbidities [1]. Here, we report a multidisciplinary management of ECAA near the skull base involving head and neck surgery (HNS), vascular surgery (VS), and neuro-interventional radiology (NIR).

## Case presentation

**Figure 1:**
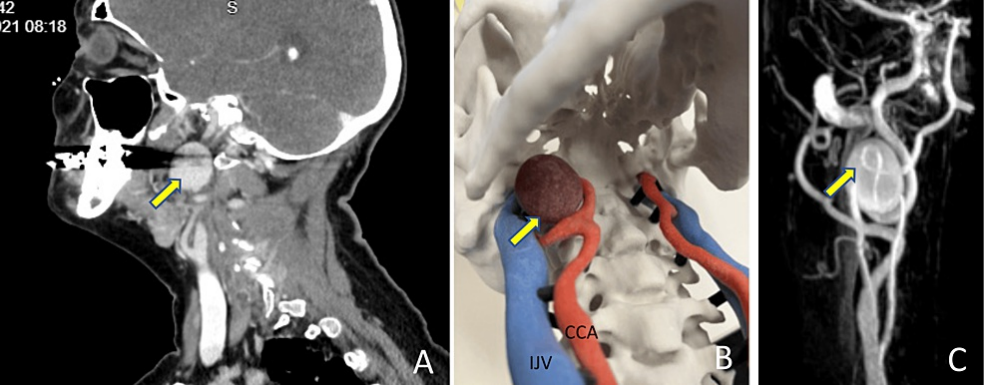
Preoperative imaging and 3D model (A) Sagittal view of CT scan demonstrating ECAA; (B) 3D model showing ECAA; (C) MRA showing aneurysm and its connection to ICA CCA: common carotid artery; IJV: internal jugular vein, yellow arrow: ECAA

A 59-year-old female was referred to vascular and head and neck surgeons with an exam finding of a subtle mass in the right upper neck with prominent pulsation. CT neck with contrast was obtained, demonstrating a 2.6 cm right internal carotid artery (ICA) at the level of the upper right cervical ICA, posterior to the mandible in proximity to the skull base (Figure 1A). As part of preoperative planning, time of flight (TOF), contrast-enhanced magnetic resonance angiogram (MRA) (Figure 1C), and 3D skull model were obtained for preoperative planning (Figure 1B).

The patient then underwent a preoperative balloon occlusion test (BOT) of the right ICA. The results demonstrated near symmetric filling angiographically suggesting tolerance, and the patient did not experience any neurological deficits.

**Figure 2:**
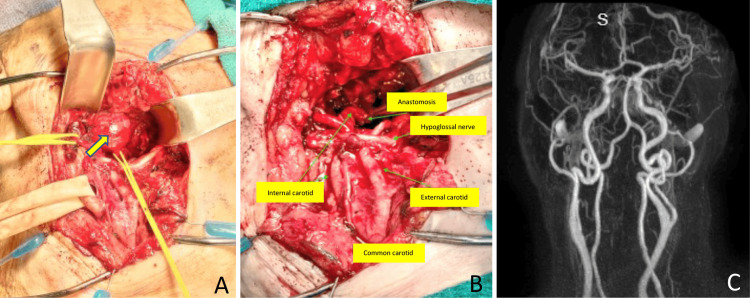
Intraoperative view of ECAA before and after surgical excision (A) ECAA (yellow arrowhead); (B) View of ECAA and anastomosis; (C) Six-month postoperative MRA showing good vascular flow in the right ICA ECAA: extracranial carotid artery aneurysm; ICA: internal carotid artery

The patient underwent excision of the right ICA aneurysm via the transcervical approach followed by end-to-end anastomosis with a great saphenous vein (GSV) graft (Figures 2A, 2B). Postoperatively, the patient developed right soft palate palsy, which resolved in a couple of months. The six-month postoperative MRA showed good vascular flow in the right ICA (Figure 2C).

## Discussion

ECAAs are classically defined as an increase in the diameter of the original arterial lumen by more than 50% [1]. Although the exact pathogenesis of ECAA is unknown, chronic inflammation with pro-inflammatory cytokines and matrix metalloproteinases and the loss of smooth muscle cells in the tunica media layer of arteries may underlie the development of an aneurysm [6].

Attigah et al. classified ECAA into five types based on anatomical location [7]. Type I is defined as isolated and short aneurysms located above the carotid bulb, type II is a long aneurysm from the carotid bulb up to the line between the mastoid process and the angle of the mandible, type III is an aneurysm of the proximal ICA and the carotid bifurcation, type IV is an aneurysm of the ICA and common carotid artery (CCA), and type V is an isolated aneurysm of the CCA. Our patient had a Type I ECAA, as the aneurysm is isolated to the internal carotid artery. Type I is thought to be the most common ECAA and is typically reconstructed with resection and end-to-end anastomosis [7]. Both an open approach with an interposition graft and endovascular treatment with a stent graft are the mainstay of therapy for all types of ECAAs [3]. Garg et al. describe five different types of open surgical options: clipping, resection and primary end-to-end anastomosis, interposition graft, extracranial to intercranial bypass, and carotid artery ligation [8]. While endovascular techniques are gaining popularity in treating various types of ECAA due to their noninvasive nature, Attigah et al. describe a 20% risk of cerebral embolism from coiling material and thrombus in the aneurysm sac [7]. With open surgery, the reported focal neurological complication rate ranges between 3% and 17% [7]. Therefore, the treatment should be tailored to individual patients. Our patient elected to undergo open repair with GSV for favorable durability.

Multidisciplinary preoperative planning is key to the successful management of ECAA, especially ones that exist near the skull base. In addition to maintaining sufficient cerebrovascular circulation, minimizing stroke risk, and preventing hemorrhage, a unique challenge for aneurysms near the skull base is proper exposure for vessel control [9]. To optimize surgical success, we designed a 3D skull model preoperatively to better visualize the location of ECAA. Moreover, the 3D skull model provides the patient with an improved understanding of the pathology. It also plays an important role in the treatment discussion. NIR performed a balloon occlusion test to ensure the option of sacrificing the carotid artery in the event of significant bleeding. HNS and VS planned and executed a transcervical approach to the ECAA and end-to-end anastomosis with GSV, respectively. Given the potential for catastrophic complications, multidisciplinary efforts and careful preoperative planning are key to successful surgical outcomes.

## Conclusions

Given the rarity of ECAAs, few guidelines exist regarding the optimal choice between medical management, endovascular interventional, and open surgery. We present a multidisciplinary approach to a near skull base ECAA by having a balloon occlusion test with NIR, GSV graft with vascular surgery, and excision via a transcervical approach with HNS. To have proper vessel exposure and minimize the risk of stroke, hemorrhage, and insufficient cerebrovascular perfusion, we recommend incorporating these multidisciplinary efforts with meticulous preoperative planning.
